# Characterizing early child growth patterns of height-for-age in an urban slum cohort of Bangladesh with functional principal component analysis

**DOI:** 10.1186/s12887-017-0831-y

**Published:** 2017-03-21

**Authors:** Yin Zhang, Jianhui Zhou, Feiyang Niu, Jeffrey R. Donowitz, Rashidul Haque, William A. Petri, Jennie Z. Ma

**Affiliations:** 1 Department of Statistics, University of Virginia, Charlottesville, VA USA; 2 Division of Pediatric Infectious Diseases, Children’s Hospital of Richmond at Virginia Commonwealth University, Richmond, VA USA; 3 International Centre for Diarrhoeal Disease Research, Bangladesh (ICDDR, B), Dhaka, Bangladesh; 4 Division of Infectious Diseases and International Health, Department of Medicine, University of Virginia, Charlottesville, VA USA; 5 Department of Public Health Sciences, University of Virginia, P.O. Box 800717, Charlottesville, 22908 VA USA

**Keywords:** Anthropometry, Longitudinal growth, Stunting, Growth faltering, Functional data analysis

## Abstract

**Background:**

Early childhood is a critical stage of physical and cognitive growth that forms the foundation of future wellbeing. Stunted growth is presented in one of every 4 children worldwide and contributes to developmental impairment and under-five mortality. Better understanding of early growth patterns should allow for early detection and intervention in malnutrition. We aimed to characterize early child growth patterns and quantify the change of growth curves from the World Health Organization (WHO) Child Growth Standards.

**Methods:**

In a cohort of 626 Bangladesh children, longitudinal height-for-age z-scores (HAZ) were modelled over the first 24 months of life using functional principal component analysis (FPCA). Deviation of individual growth from the WHO standards was quantified based on the leading functional principal components (FPCs), and growth faltering was detected as it occurred. The risk factors associated with growth faltering were identified in a linear regression.

**Results:**

Ninety-eight percent of temporal variation in growth trajectories over the first 24 months of life was captured by two leading FPCs (FPC1 for overall growth and FPC2 for change in growth trajectory). A derived index, adj-FPC2, quantified the change in growth trajectory (i.e., growth faltering) relative to the WHO standards. In addition to HAZ at birth, significant risk factors associated with growth faltering in boys included duration of breastfeeding, family size and income and in girls maternal weight and water source.

**Conclusions:**

The underlying growth patterns of HAZ in the first 2 years of life were delineated with FPCA, and the deviations from the WHO standards were quantified from the two leading FPCs. The adj-FPC2 score provided a meaningful measure of growth faltering in the first 2 years of life, which enabled us to identify the risk factors associated with poor growth that would have otherwise been missed. Understanding faltering patterns and associated risk factors are important in the development of effective intervention strategies to improve childhood growth globally.

**Trial registration:**

ClinicalTrials.gov Identifier: NCT02734264, registered 22 March, 2016.

**Electronic supplementary material:**

The online version of this article (doi:10.1186/s12887-017-0831-y) contains supplementary material, which is available to authorized users.

## Background

The early years of life are important for physical growth and brain development. The World Health Organization (WHO) growth standards for infants and young children can be used to identify children who are at risk of growth faltering, including stunting and wasting [1, 2] (http://www.who.int/childgrowth/mgrs/en/), (http://www.who.int/nutgrowthdb/about/introduction/en/index2.html). Stunting and wasting, defined as height-for-age (HAZ) and weight-for-height (WHZ) below 2 standard deviations of the WHO standards, are usually consequences of malnutrition and other health problems (http://www.who.int/childgrowth/mgrs/en/) [3]. Malnutrition and stunting are prevalent in developing countries and cause substantial childhood morbidity and mortality [2]. Nearly one-third of the children in developing countries are undernourished by the age of 15 months. Intrauterine growth restriction, stunting, and severe wasting before age 5 years cause 2.2 million annual deaths and 21% of all disability-adjusted life-years [2, 4]. Morbidity from malnutrition before age 5 years affects physical growth and cognitive development in 200 million children, including 86 million children in India subcontinent [2, 4]. Young children who do not meet their full potential for physical and cognitive development are at greater risk of poor health and poverty in adulthood [3, 5, 6], and this perpetuates the vicious cycles of poverty and impaired development [7, 8]. Therefore, it is important to characterize growth patterns in early childhood and identify the determinants associated with growth faltering to facilitate screening strategies and development of effective interventions [2, 9, 10].

Although childhood growth is a continuous process, growth measurements are typically collected at discrete times [10]. Traditional studies on growth and nutrition often considered growth measures at a single or few time points as the responses. For example, change in HAZ from birth is often used to measure whether a child growth is healthy or not, but this quantity may be imprecise because different growth patterns can yield a similar change in HAZ. Functional data analysis (FDA) methods that model observed discrete data with continuous underlying process would be more appropriate. FDA focuses on data that represent infinite-dimensional and continuous process such as curves, shapes, and images, and has broad potential application to biomedical research fields [11, 12]. Specifically, the functional principal component analysis (FPCA) [13–16], one of the commonly used FDA methods, is useful to characterize childhood growth patterns. FPCA performs dimension reduction for functional data by identifying dominant modes of variation and extracting several uncorrelated and ordered principal components. We applied FPCA to HAZ over the first 24 months of life to characterize early childhood growth patterns in a birth cohort of Bangladeshi children. Although growth faltering is widely used synonymously with “failure to thrive”, to our knowledge no consensus exists regarding its specific definition [17–21] (http://www.pmh.health.wa.gov.au/general/CACH/docs/manual/3%20Birth%20to%20School%20Entry/3.4/3.4.2%20Growth%20faltering.pdf) [22]. Because characterizing the growth pattern in HAZ is of primary interest in this study, growth faltering here is defined as slower-than-expected growth in HAZ according to the WHO Child Growth Standards; i.e., downward deviation in attained HAZ. We aimed to quantify such deviation of growth from the WHO reference and identify its associated risk factors.

## Methods

### Study cohort

This was a prospective cohort study conducted between January 1, 2008 and December 31, 2012 in Dhaka, Bangladesh, consisting of 330 boys and 296 girls who lived in an urban slum of the Mirpur Thana area. Potential study subjects were identified from a local census for pregnant women, and healthy newborns were enrolled within 72 h of birth after written informed consent from the parents or guardians. Infants were followed until 2 years old. Information about socioeconomic status, maternal health, and hygienic practice was collected at enrollment. Maternal height (m) and weight (kg) were also measured at enrollment. Follow-up and surveillance were performed by trained research staff members who visited each study house twice a week and recorded information about child morbidity using a structured questionnaire. When a child had an acute illness, he or she was referred to the study clinic for further evaluation and treatment. Information on exclusive breast-feeding was obtained from monthly reports about the child’s consumption of human milk without supplementation (including water but excluding medications), and breast-feeding practices were monitored by field observation throughout infancy. Details about the study population and surveillance were described previously [9, 23–25]. The study was approved by the Institutional Review Board of the University of Virginia and the Ethical Review Committee of the International Centre for Diarrhoeal Disease Research, Bangladesh (icddr,b).

### Anthropometric responses

Anthropometric measurements in this observational cohort study were collected by field research assistants upon enrollment and every 3 months during follow-up. All research staff were trained in appropriate techniques for anthropometry measurement and nutritional status assessment in a community setting. Weight and length of the children were measured with electronic scales and length boards that were precise to 0.01 kg and 0.1 cm (SECA Gmbh & Co, Hamburg, Germany). All anthropometry measurement instruments were well maintained and calibrated. The mean of two consecutive measurements at each visit was recorded. Nutritional status was assessed by comparing height and weight in the study cohort with the WHO growth reference for the same age and sex (WHO Anthro software, version 3.0.1, WHO, Geneva, Switzerland) [9]. Specifically, height-for-age z-score (HAZ) is an age- and sex-normalized measure of child height given in units of standard deviations and relative to the median age- and sex-conditional height distribution of the WHO reference population [26, 27]. A positive (+) or negative (−) sign depends on whether the child’s actual height is more or less than the median height of the WHO reference population at that age for that sex. When the actual height of a child is exactly equal to the median, the resultant HAZ is 0 (zero). The HAZ is of particular interest because it captures the long-term cumulative effects of health throughout childhood and is known to be correlated with later life outcomes [3, 28]. In this study, longitudinal HAZs in the first 2 years of age were the response measurements. For reliable estimation of individual growth curves, only those children who had ≥ 5 anthropometric measurements (i.e., who had growth observations approximately for the first year) were included.

### Functional principal component analysis for growth curves

Considering a continuous underlying growth curve of HAZs for each child, the directional change in growth and variation among growth curves of the cohort were identified and captured with the functional principal component analysis (FPCA) [11]. Separate FPCA was performed for boys and girls because of the effect of sex on growth and development. The FPCA estimated dominant modes of temporal variation among the individual growth, and extracted leading functional principal components (FPCs) that represented the temporal patterns associated with the largest proportions of variation. Each individual growth curve of HAZ was then approximated by summation of the estimated mean curve and a linear combination of the leading FPCs. The coefficients of these FPCs were referred to as the FPCA scores, characterizing the deviation of the individual curve from the mean curve.

There were several challenges in the growth modeling of HAZ in this study with traditional FPCA [11]. The HAZ growth data were relatively sparse, and measured at different times. Some data were incomplete for the entire 2 years because of early dropout. In addition, observations from the growth curves were subject to measurement error. To overcome those challenges, we performed the FPCA method using “funeigen” in the “funreg” package (version 1.1) in R 3.1 (http://cran.r-project.org/web/packages/funreg/index.html), which is able to accommodate sparse unbalanced data and account for measurement error [29]. This method was similar to the principal analysis through conditional expectation (PACE) method [12].

### Quantifying downward change in HAZ

**Fig. 1:**
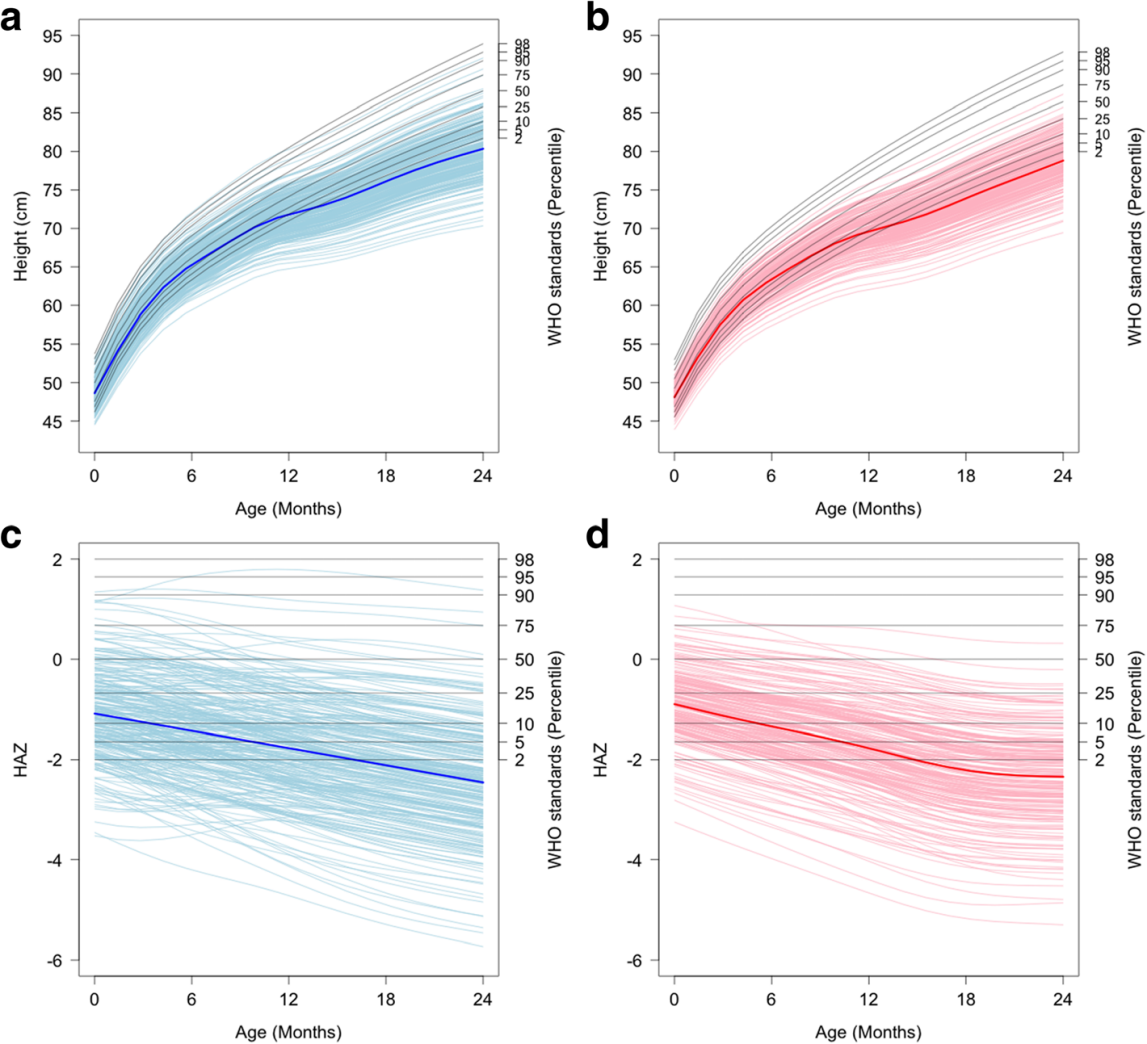
Individually fitted curves of height (Panels **a** and **b**) and height-for-age z-score (HAZ) (Panels **c** and **d**) from birth to age 24 months for boys (*light blue*, *n* = 270) and girls (*pink*, *n* = 225). The mean *curves* are shown in *solid blue line* for boys and *red line* for girls. The World Health Organization (WHO) growth standards are shown as the *solid black lines* in Panels **a** and **b** and as *solid horizontal black lines* in Panels **c** and **d** for the WHO “pseudo children” who were growing along the WHO percentiles, separately for boys and girls

The fitted growth curves from PFCA in actual height (cm) and HAZ for boys and girls were plotted with the WHO growth standards that described the sex-specific percentiles of childhood growth from birth to age 24 months (Fig. 1). The fitted curves for most children in the cohort were below the 50th percentile of the WHO growth standards and deviated further down from the standards with increasing age, i.e., most subjects had downward growth patterns in HAZ with respect to the WHO growth standards [1] (http://www.who.int/childgrowth/mgrs/en/) [27]. With FPCA, these fitted individual growth trajectories can be characterized by the leading FPCs and the corresponding FPC scores measure the deviation of individual curves from the mean curve. However, these FPC scores are only relative within the study cohort. To measure the downward growth comprehensively from the expected with respect to the WHO growth standards, we derived an index based on the FPC1 and FPC2 scores along with the WHO standards as described below.

For each subject in the study cohort, we defined a reference curve from the WHO growth standards and quantified downward growth trajectories of HAZ based on the deviation of the subject’s growth curve from the corresponding WHO reference curve. We first estimated the FPC1 and FPC2 scores for those “pseudo children” who had exact growth along the available WHO percentiles and defined them as the reference FPC scores. These reference FPC scores were estimated by regressing the difference between the reference and estimated mean curves on the two FPCs evaluated over 18 equally spaced times from birth to age 24 months. These reference FPC1 scores were used to classify subjects into 5 FPC1 strata that were defined by two consecutive reference FPC1 scores in the order of percentiles of those “pseudo children”. A similar classification method for functional data using FPC scores was developed previously [30]. Each stratum consisted of children who had similar growth patterns, and the WHO standard curve that corresponded to the upper limit of the stratum served as the reference curve for all subjects in that stratum. Then, within a given FPC1 stratum, an adjusted FPC2 score (adj-FPC2) for a child was calculated as the difference between the subject’s FPC2 score and the FPC2 score of the reference curve; i.e., adj-FPC2 = child’s FPC2 score - FPC2 score of the corresponding WHO reference curve. Because the adj-FPC2 score accounted for the FPC1 score indirectly through stratification and for the FPC2 score directly in the calculation, it measured deviation of individual growth trajectory from the expected with respect to the WHO standards, and thus quantified the growth faltering from the WHO reference. A positive adj-FPC2 indicated that the child had downward growth in HAZ relative to the corresponding WHO reference curve. The larger a positive adj-FPC2, the greater the change in growth or the more severe the growth faltering.

### Risk factor analysis

After the adj-FPC2 score was calculated to quantify growth faltering, we considered it as the response and evaluated its associated risk factors in a linear regression model. Similar idea of using FPC scores as predictors or responses in the subsequent analysis has been proposed in the literature [31]. For interpretation purposes, the continuous risk factors were centered at the mean in the regression analysis. Risk factors of interest included HAZ at birth, maternal height and weight, mother with any formal education, family size, monthly family income, duration of exclusive breast-feeding, number of diarrheal episodes from birth to age 6 months, source of drinking water, food coverage practice, and strata of FPC1 score. We also calculated the false discovery rate (FDR) adjusted *p*-values for these risk factors using the Benjamini and Hochberg method [32].

## Results

Of the 626 children enrolled in the original birth cohort, 495 (270 boys and 225 girls) had ≥ 5 anthropometric measurements and were included in this study (Additional file 1: Figure S1). As shown in Table 1, the HAZ at birth for the total cohort was at −0.94 ± 1.14 with 18% of the boys and 14% of the girls being stunted. The socioeconomic status of the study cohort was low; approximate 37% of the mothers never had any formal education, and the average monthly family income was below 7000 Bangladesh taka (approximately $90 US dollars). Four percent of the children were born pre-term. The majority of households had access to municipal water supplies and employed food coverage practices. Six percent of families had an animal in the house, and 35% of the households had access to septic tanks or toilets. These children were exclusively breastfed for 4 months on average. Fifty-one percent of the children had experienced ≥ 2 diarrheal episodes from birth to age 6 months (Table 1).

**Table 1:** Characteristics and risk factors of the study subjects^a^

Characteristic/risk factor	Boys (*n* = 270)	Girls (*n* = 225)	Total (*n* = 495)
HAZ at birth	−1.00 ± 1.14	−0.88 ± 1.13	−0.94 ± 1.14
Maternal height (cm)	149.91 ± 5.76	149.47 ± 5.14	149.71 ± 5.48
Maternal weight (kg)	48.12 ± 8.50	47.92 ± 8.36	48.03 ± 8.43
Monthly family income (1000 Bangladesh taka)^b^	6.95 ± 3.15	6.97 ± 3.99	6.96 ± 3.55
Mother with any formal education (%)	172 (63.7)	142 (63.1)	314 (63.4)
Family size ≥ 5 (%)	163 (60.4)	136 (60.5)	299 (60.4)
Preterm birth (<37 weeks, %)	10 (3.7)	10 (4.4)	20 (4.0)
Drinking water from municipality supply (%)	260 (96.3)	217 (96.4)	477 (96.4)
Food coverage practiced at household (%)	260 (96.3)	215 (95.6)	475 (96.0)
Having an animal in the house (%)	22 (8.2)	8 (3.6)	30 (6.1)
Access to a septic tank/toilet (%)	109 (40.4)	66 (29.3)	175 (35.4)
Duration of exclusive breast-feeding (month)	4.09 ± 2.33	4.07 ± 2.16	4.08 ± 2.25
≥2 diarrheal episodes in first 6 months (%)	138 (51.1)	112 (49.8)	250 (50.5)

*Abbreviations*: *HAZ* height-for-age z-score

^a^Data reported as mean ± SD for continuous measures and number (%) for categorical variables

^b^7000 Bangladesh taka = approximately $90 (United States)

### Functional principal component analysis to quantify downward change in HAZ

**Fig. 2:**
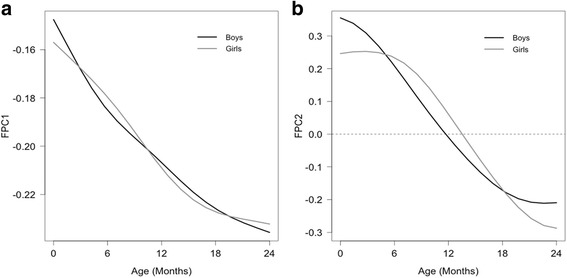
The two leading functional principal components (FPCs) in boys (*black*) and girls (*grey*). The FPC1 in both boys and girls (Panel **a**) were *negative* and monotonically *decreased* over time, reflecting further deviation of individual growth patterns from the mean *curve*. The FPC2 (Panel **b**) changed signs approximately at 12 months for boys and 14 months for girls, indicating the changes in growth trajectory. For boys, the FPC1 and FPC2 accounted for 93 and 6% of the variation among fitted height-for-age z-score (HAZ) *curves*. For girls, the FPC1 and FPC2 accounted for 96 and 3% of the variation among fitted HAZ *curves*

The essential modes of temporal variation among the fitted curves were extracted by FPCs. The two leading FPCs (FPC1 and FPC2) accounted for > 98% of the temporal variation in the HAZ growth trajectories: with 93% in FPC1 and 6% in FPC2 for boys and 96% in FPC1 and 3% in FPC2 for girls (Fig. 2). The FPC1 and FPC2 captured the directions of the departure of individual curves from the mean curve. The FPC1 decreased with increasing age in boys and girls, consistent with progressively decreased HAZ. The FPC2 also decreased with age but was positive at a younger age and negative at an older age, implying a directional change in the growth trajectory with respect to the mean curve.

**Fig. 3:**
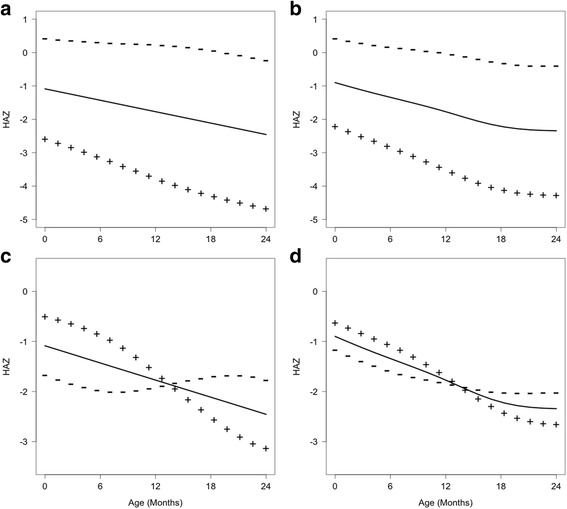
Functional principal component analysis (FPCA) results for boys (Panels **a** and **c**) and girls (Panels **b** and **d**). The mean *curves* for height-for-age z-score (HAZ) are shown as *solid lines*, and the changes from the mean *curves* for FPC1 (Panels **a** and **b**) and FPC2 (Panels **c** and **d**) are shown in “+++” and “---” when 2•SD (*standard deviation*) of FPC scores are added to (the “+++” *curve*) or subtracted from (the “---” *curve*)

**Fig. 4:**
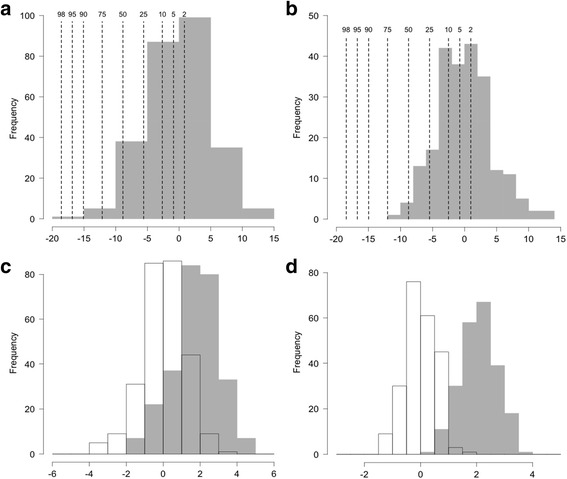
Histograms of estimated FPC1 scores (*solid bars* in Panels **a** and **b**), original FPC2 scores (*clear bars* in Panels **c** and **d**) and adj-FPC2 scores (*solid bars* in Panels **c** and **d**) in boys and girls. The FPC1 scores for WHO “pseudo children” (growing along WHO percentiles) are shown as *dashed lines* in Panels **a** and **b**, whereas most study children were below the WHO 50^th^ percentile. *Positive* adj-FPC2 scores in Panels **c** and **d** quantified the degree of growth faltering. *Abbreviations*: adj-FPC2, adjusted FPC2; FPC, functional principal component; WHO, World Health Organization

Plots of the fitted mean curves of HAZ vs age and the curves that were 2 standard deviations of the FPC scores above (+++) and below (---) the mean curves showed a broad range of HAZ growth curves along with FPC scores (Fig. 3). For FPC1, the curves that were 2 standard deviations of the FPC1 scores above and below the mean curves had similar patterns as the mean curve (Fig. 3a and b), which captured the decreasing growth and were interpreted as *overall growth pattern*; a larger positive FPC1 score indicated an accelerated decreasing curve, and a negative FPC1 score indicated a slower decreasing curve of HAZ in relation to mean curve. In contrast, for FPC2, the curves that were 2 standard deviations of the FPC2 scores above and below the mean curve crossed over the mean curve at approximately 12 months of age for both boys and girls (Fig. 3c and d) and thus captured *the change in growth trajectory*; a larger positive FPC2 score indicated a greater change in growth trajectory relative to the mean curve. However, the FPC2 alone was not very informative to quantify the degree of downward growth or growth faltering relative to the WHO standards, as the FPC2 score was centered at a mean zero for the cohort in the FPCA method, which would imply roughly 50% of children in the cohort had positive/negative FPC2 scores. In other words, the FPC2 scores measured the relative growth faltering by comparing the subjects within the cohort, not to the WHO growth standards. Comparatively, the unadjusted FPC2 scores for most subjects were larger than the reference FPC2 scores of the WHO standard curves, and the adjustment for the WHO standards and stratification on FPC1 scores resulted in that adj-FPC2 scores were positive for all children except 29 boys (Fig. 4). The results were consistent with the growth patterns observed in Fig. 1, where most children in the cohort had downward growth in HAZ. Therefore, using the WHO standards as the benchmark, the adj-FPC2 was a meaningful index to quantify the degree or severity of growth faltering as it accounted for the magnitude and direction of deviation in growth trajectory from the WHO standards.

### Risk factors associated with growth faltering

As described in the Methods Section, children were classified into five strata on the basis of their FPC1 scores. Children in each stratum had similar overall growth patterns. The mean adj-FPC2 score was the highest in Stratum 1 and the lowest in Stratum 5 (Table 2), indicating an ordered degree of growth faltering, with the most severe faltering seen in Stratum 1. The linear regression analysis of the adj-FPC2 score on the risk factors and the FPC1 strata showed that overall model fitting was adequate (*R* ^2^ = 0.64 for boys and 0.63 for girls) (Table 3). Significance of FPC1 strata suggested that the overall growth was important for growth faltering. Children with poorer overall growth (lower FPC1 strata) were more likely to have growth faltering (Table 3). HAZ at birth was significant for boys and girls without and with FDR adjustment, but because of the high correlation between HAZ at birth and FPC1 score this needed to be interpreted with FPC1 stratum. Larger babies at birth were more likely to experience poorer growth, especially for those in lower strata, and the effect was attenuated for those in higher strata. Such an observation could be in part due to the regression to the mean.

**Table 2:** Descriptive summary of the adj-FPC2 score stratified by FPC1 score according to the WHO growth standards^a^

		Boys		Girls	
Stratum	WHO Standard (percentile)	No. of Subjects (%)	adj-FPC2^b^	No. of Subjects (%)	adj-FPC2^b^
Stratum 1	2	117 (43.3)	2.04 ± 1.21	92 (40.9)	2.33 ± 0.58
Stratum 2	5	41 (15.2)	1.92 ± 0.98	36 (16.0)	2.23 ± 0.58
Stratum 3	10	41 (15.2)	1.49 ± 1.28	38 (16.9)	2.09 ± 0.48
Stratum 4	25	39 (14.4)	1.68 ± 1.16	36 (16.0)	1.61 ± 0.45
Stratum 5^c^	50-98	32 (11.9)	1.04 ± 1.22	23 (10.2)	1.47 ± 0.61

*Abbreviations*: *adj-FPC2* adjusted FPC2 score, *WHO* World Health Organization

^a^Data reported as mean ± SD or number (%)

^b^Adj-FPC2 score in each stratum was calculated as the difference of FPC2 score and corresponding stratum-specific WHO reference FPC2 score, where strata were defined based on the magnitude of FPC1 score with respect to the WHO reference FPC1 score

^c^Stratum 5 represents children who were classified into the strata of 50th to 98th percentiles because of the few children in these strata

**Table 3:** Risk factors associated with growth faltering measure (adj-FPC2 score) from linear regression, separately for boys and girls^a^

	Boys			Girls		
Risk factor	Coefficient	*p*-value	Adj. *p*-value^c^	Coefficient	*p*-value	Adj. *p*-value^c^
(Intercept)	3.392	<0.0001	–	3.692	<0.0001	–
HAZ at birth	0.945	<0.0001	<0.0001	0.402	<0.0001	<0.0001
Maternal height (cm)	−0.004	0.7246	0.7672	0.011	0.0743	0.1486
Maternal weight (kg)	−0.007	0.3314	0.4780	−0.008	0.0327	0.0840
Income (1000 Bangladesh taka)	−0.047	0.0086	0.0221	−0.012	0.2090	0.3134
Mother with any formal education	−0.076	0.4656	0.5717	0.006	0.9280	0.9280
Family size ≥ 5	0.273	0.0101	0.0226	0.012	0.8428	0.9280
Preterm birth (<37 weeks)	0.180	0.4764	0.5717	−0.202	0.1332	0.2180
Household water supply from municipality	−0.328	0.1962	0.3531	−0.411	0.0062	0.0185
Food coverage practiced at household	0.237	0.3452	0.4780	−0.262	0.0559	0.1257
Having an animal in the house	−0.353	0.0402	0.0804	−0.082	0.5801	0.6961
Access to a septic tank/toilet	−0.096	0.3283	0.4780	−0.008	0.9024	0.9280
Duration of exclusive breast-feeding ≤ 6 months	0.013	0.6680	0.7515	−0.014	0.4498	0.5784
Duration of exclusive breastfeeding > 6 months	0.056	0.0071	0.0213	−0.015	0.2602	0.3603
≥2 diarrheal episodes in first 6 months	0.009	0.9243	0.9243	−0.094	0.0980	0.1764
Stratum 2	−0.570	0.0002	0.0006	−0.312	0.0003	0.0009
Stratum 3	−1.033	<0.0001	<0.0001	−0.694	<0.0001	<0.0001
Stratum 4	−1.553	<0.0001	<0.0001	−1.118	<0.0001	<0.0001
Stratum 5^b^	−2.602	<0.0001	<0.0001	−1.641	<0.0001	<0.0001

*Abbreviations*: *HAZ* height-for-age z-score

^a^ *R* ^2^ = 0.64 for boys and *R* ^2^ = 0.63 for girl

^b^Strata were defined based on the FPC1 score with respect to the WHO reference FPC1 score. Stratum 5 represents children who were classified into the strata of 50th to 98th percentiles because of the few children in these strata

^c^Adjusted *p*-value corrected for false discovery rate (FDR) using the Benjamini and Hochberg method (1995)

In addition, without FDR adjustment, significant factors associated with the poor growth (*p*-value < 0.05) were family income, family size, having an animal in the house and exclusive breastfeeding over 6 months for boys, and maternal weight and access to municipal water supplies for girls. Specifically, boys from higher-income families or having an animal in the house were less likely to have growth faltering, whereas those from larger families and exclusively breastfed for longer than 6 months were more likely to have poor growth. Girls were less likely to have faltering if their mothers had normal weight or their households had access to the municipal water supply. Without adjustment, food coverage practice had a marginally significant effect against faltering in girls (*p*-value = 0.056). After FDR adjustment, these identified risk factors remained significant or marginally significant, except for the food coverage practice in girls.

Table 4 shows the Pearson correlations of FPC scores from FPCA with HAZs and the changes in HAZ percentiles over time. Note that HAZs represented overall growth trajectory and the change in HAZ percentiles reflected relative change in growth trajectory. The FPC1 score was significantly correlated with HAZ at birth, ages 6, 12, 18, and 24 months (Pearson correlation coefficient: boys, −0.996 to −0.849; girls, −1.00 to −0.971). However, the FPC1 score was only weakly or not correlated with the changes of HAZ percentiles from birth to age 24 months or from 6 to 18 months (Table 4). These results agreed with the interpretation that FPC1 captured the overall growth pattern but not the change of the pattern. In contrast, the adj-FPC2 score was highly correlated with the changes of HAZ percentiles over time, but only weakly with individual HAZ measurements (Table 4). These results agreed well with the notion that adj-FPC2 captured the downward change in the growth trajectory, i.e., growth faltering. Moreover, FPC1 and adj-FPC2 scores would be better summary indices than individual HAZ measurements at discrete times because they together characterized the entire growth process continuously from birth to age 2 years.

**Table 4:** Correlations between HAZ measurements and functional principal component scores: FPC1 score and adj-FPC2 score^a^

HAZ or HAZ Difference	Boys		Girls	
	FPC1 Score ^b^	Adj-FPC2 Score^c^	FPC1 Score^b^	Adj-FPC2 Score^c^
HAZ at enrollment	−0.849	0.292	−0.971	−0.136^d^
HAZ at 6 months	−0.940	0.100^e^	−0.982	−0.179^d^
HAZ at 12 months	−0.996	−0.304	−1.000	−0.350
HAZ at 18 months	−0.980	−0.427	−0.992	−0.459
HAZ at 24 months	−0.979	−0.425	−0.990	−0.467
Change of HAZ %tiles from birth to age 24 months	−0.156^d^	−0.919	−0.047^e^	−0.794
Change of HAZ %tiles between age 6 and 18 months	−0.062^e^	−0.905	−0.028^e^	−0.787

*Abbreviations*: *HAZ* height-for-age z-score, *adj-FPC2* adjusted FPC2 score

^a^Reported as Pearson correlation coefficient

^b^FPC1 score was highly correlated with HAZs over times (*p*-value < 0.0001), but not correlated with the change of HAZ percentiles (%tile) from birth to age 24 months and that between 6 and 18 months in both boys and girls

^c^Adj-FPC2 was highly correlated with the change of HAZ percentiles (%tile) from birth to age 24 months and that between 6 and 18 months (*p*-value < 0.0001), but only weakly or not correlated with HAZs over times in both boys and girls

^d, e^All the correlations were significantly different from zero with *p*-value < 0.0001, except those indicated as ^4^ 0.008 < *p*-value < 0.05 and ^5^ *p*-value > 0.1

## Discussion

In this study, the physical growth patterns in an urban slum cohort of Bangladeshi children from birth to age 2 years were modeled with the FPCA method. The variation in growth trajectories of HAZ for both boys and girls were characterized by two leading FPCs, where FPC1 captured the overall growth pattern and FPC2 captured the change in growth trajectory from birth to age 2 years. The adj-FPC2 was derived from stratified FPC1 score and individual FPC2 score coupled with the WHO growth standards to quantify growth faltering relative to the WHO standards. Most children in the study had growth faltering as shown in Fig. 1. Also, as shown in Fig. 4 and Table 4, the adj-FPC2 was a more meaningful index for quantifying growth faltering than the original FPC2 and the individual HAZ measurements. Furthermore, the adj-FPC2 can be used as a response to identify risk factors associated with growth faltering (Table 3). The present method of stratifying FPC1 scores was analogous to distance-based clustering method on functional curves previously described in the literature [30].

Growth faltering is a widely used concept, regarded as an indicator of physical or physiological problems in early childhood, and is associated with subsequent growth delay and cognitive deficiencies [18, 22, 33]. Children who experienced faltering in infancy weighed less and were shorter at school age, with adverse intellectual outcomes [5, 34]. However, no consensus exists about its specific definition or quantitative measurement. In this study, we attempted to use a single index (i.e., adj-FPC2 score) derived from FPCA results and the WHO standards to describe growth faltering quantitatively from birth to age 2 years. Indeed, the FPCA results enabled us to investigate the major growth patterns closely and quantify the growth change or faltering from a different viewpoint. In addition, our approach can be applied to other longitudinal measurements in early childhood such as weight and cognitive scores to quantify faltering in these measures as well. It is our overarching goal to derive an index that does not only quantify the deviation of individual growth from the WHO reference but also is comparable across different cohorts.

Causes of childhood growth faltering are multifactorial, including inadequate food and nutrient intake, frequent infections, child feeding behaviors, sanitation measures, environmental enteric dysfunction, and genetics [35]. An important contribution of this study was the delineation of potential risk factors associated with the adj-FPC2 score for measuring changes in growth patterns or faltering. Interestingly, risk factors for such faltering for the first 2 years of life appeared to be different for boys and girls. For boys, significant factors included HAZ at birth, income, family size, having an animal in the house, and exclusive breastfeeding; whereas for girls, the factors included maternal weight, access to municipal water supply, and employment of food coverage practice. The significant risk factors specific to boys or girls might in part reflect the underlying physiological difference in gender, or potential cultural difference in raising boys and girls. Our findings were consistent with the literature [36, 37]. Mother’s education was found to be a strong predictor in a Kenyan study [38], but it was not significant in our analysis, possibly due to its confounding effect with other risk factors. Significant effects of FPC1 strata and HAZ at birth within each stratum may imply epigenetic factors or prenatal nutrition. Although it is impossible to intervene in all the factors, some factors, such as food coverage practice, breastfeeding, water access, sanitation measures, and prenatal nutrition, are readily modifiable. These results, while informative, need to be evaluated in independent studies to confirm the importance of the first 2 years of life as a critical window within which linear growth is most sensitive to modifiable environmental factors [18, 39].

Traditional studies on growth and nutritional outcomes often considered HAZ at a single or a few time points as responses, but this captured only partial growth at these discrete times. For example, the change in HAZ from birth only accounted for the growth measures at two time points, and many different growth trajectories can yield an identical change in HAZ. In contrast, the growth patterns based on our FPCA results reflected the entire continuous growth process in the first 2 years of life, and thus are more comprehensive. In addition, using adj-FPC2 as a response identified novel risk factors. Particularly, duration of breastfeeding in boys and maternal weight and water source in girls would have been missed (data not shown) if HAZ at 2 years or change in HAZ from birth to 2 years were considered as the response.

One potential limitation of the present growth modeling with FPCA was that the results and interpretation are data dependent. Even though FPCA may decompose the variation among individual curves into uncorrelated temporal features and provide leading FPCs, the present interpretation of the FPCs may be applicable only to functional data with similar temporal variation patterns such as growth curves of weight, height, or cognitive measurement but may not be appropriate for different types of functional data such as imaging. In addition, although FPCA may be applied to sparse data, we limited the present FPCA analysis to those children who had ≥ 5 observations for reliable estimation of the individual curves. Such a practice may have resulted in potential power reduction because approximately 21% children who had < 5 observations were excluded from the analysis.

## Conclusions

The continuity of the underlying growth curves of HAZ and faltering patterns in the first 2 years of life were delineated with FPCA, and the deviations from the WHO standards in infants from the urban slum cohort were quantified from the two leading FPCs. The derived adj-FPC2 quantified the change in individual growth and provided a meaningful measure of growth faltering in the first 2 years of life relative to the WHO standards. The significant risk factors associated with poor growth were clinically plausible. With adj-PFC2, we were able to identify those risk factors associated with poor growth that would have otherwise been missed. The present findings are helpful to our understanding of early childhood growth, which can facilitate development of effective and timely prevention and/or intervention strategies to improve childhood growth globally.

## Additional file

Additional file 1: Figure S1. Online Supplemental Material. Description: Flow diagram depicting participant enrollment in this study. (DOCX 19 kb)

## Abbreviations

Adj-FPC2: Adjusted FPC2 score relative to the WHO Growth Standards
FDR: False discovery rate
FPC: Functional principal component
FPCA: Functional principal component analysis
HAZ: Height-for-age z-score
icddr,b: International Centre for Diarrhoeal Disease Research, Bangladesh
PACE: Principal analysis through conditional expectation
WHO: World Health Organization
