# Supplementary material for: Characterizing early child growth patterns of height-for-age in an urban slum cohort of Bangladesh with functional principal component analysis
Source: BMC Pediatr. 2017 Mar 21;17:84. doi: 10.1186/s12887-017-0831-y (PMC5359797; doi:10.1186/s12887-017-0831-y)
Supplement: Additional file 1: Figure S1. — Online Supplemental Material. Description: Flow diagram depicting participant enrollment in this study. (DOCX 19 kb) [file 12887_2017_831_MOESM1_ESM.docx]

**Online Supplemental Material**

Supplemental Figure 1: Flow diagram of study subject enrollment
